# Optimization of a static headspace GC-MS method and its application in metabolic fingerprinting of the leaf volatiles of 42 citrus cultivars

**DOI:** 10.3389/fpls.2022.1050289

**Published:** 2022-12-08

**Authors:** Honghong Deng, Runmei He, Rong Huang, Changqing Pang, Yuanshuo Ma, Hui Xia, Dong Liang, Ling Liao, Bo Xiong, Xun Wang, Mingfei Zhang, Xiang Ao, Bo Yu, Dongdao Han, Zhihui Wang

**Affiliations:** ^1^ Institute of Pomology and Olericulture, College of Horticulture, Sichuan Agricultural University, Chengdu, China; ^2^ Sichuan Dan Cheng Modern Fruit Industry Co., Ltd., Meishan, China; ^3^ Ningbo Tian Yuan Mu Ge Agricultural Development Co., Ltd., Ningbo, China

**Keywords:** volatile organic compounds, principal component analysis, partial least-squares discriminate analysis, mandarins (*Citrus reticulata* Blanco), orange (*Citrus sinensis* L. Osbeck)

## Abstract

Citrus leaves, which are a rich source of plant volatiles, have the beneficial attributes of rapid growth, large biomass, and availability throughout the year. Establishing the leaf volatile profiles of different citrus genotypes would make a valuable contribution to citrus species identification and chemotaxonomic studies. In this study, we developed an efficient and convenient static headspace (HS) sampling technique combined with gas chromatography-mass spectrometry (GC-MS) analysis and optimized the extraction conditions (a 15-min incubation at 100 ˚C without the addition of salt). Using a large set of 42 citrus cultivars, we validated the applicability of the optimized HS-GC-MS system in determining leaf volatile profiles. A total of 83 volatile metabolites, including monoterpene hydrocarbons, alcohols, sesquiterpene hydrocarbons, aldehydes, monoterpenoids, esters, and ketones were identified and quantified. Multivariate statistical analysis and hierarchical clustering revealed that mandarin (*Citrus reticulata* Blanco) and orange (*Citrus sinensis* L. Osbeck) groups exhibited notably differential volatile profiles, and that the mandarin group cultivars were characterized by the complex volatile profiles, thereby indicating the complex nature and diversity of these mandarin cultivars. We also identified those volatile compounds deemed to be the most useful in discriminating amongst citrus cultivars. This method developed in this study provides a rapid, simple, and reliable approach for the extraction and identification of citrus leaf volatile organic compound, and based on this methodology, we propose a leaf volatile profile-based classification model for citrus.

## Introduction

Plants have evolved an extensive variety of secondary metabolites, including biogenic volatile organic compounds (VOCs), that facilitate interactions with their surrounding environment (Ninkovic et al., 2021), from attracting mutualists such as pollinators and seed dispersers to providing protection against harmful insects, pathogens, parasites, herbivores, and environmental stresses (Bouwmeester et al., 2019). Furthermore, by interacting with plant hormones, VOCs may influence plant growth, development, and senescence (Brilli et al., 2019). To date, more than 1,700 VOCs have been identified among angiosperms and gymnosperms in 90 different plant families (Knudsen et al., 2006). Giving that plants are sessile organism, VOCs could to a certain extent be said to provide the basis for a system of plant communication (Ninkovic et al., 2021).

Citrus species are economically and nutritionally important fruit crop plants grown in more than 140 countries in tropical and subtropical regions worldwide (Cuenca et al., 2018). One of the main characteristics of these plants is the presence of aromatic essential oils in most tissues, including the leaves, stems, flowers, and fruits. These essential oils are complex mixtures that can contain hundreds of VOCs (González-Mas et al., 2019). However, although researchers have made considerable advances determining the volatile profiles of citrus fruit (Gonzalez-Mas et al., 2011; Rambla et al., 2014; Zhang et al., 2017; Zhang et al., 2019), comparatively little attention has focused on leaf VOCs.

Citrus leaves, a rich source of VOCs, have the beneficial properties of rapid growth, large biomass, and availability throughout the year (Azam et al., 2013). In addition, the volatome of citrus leaves can be used for the elucidation of complex phylogenetic relationships among taxa within the *Citrus* genus (Lamine et al., 2018), and for evaluating the metabolic changes that take place during defense responses against abiotic stresses (Matsukawa et al., 2017). In our previous study, we found that Huanglongbing (HLB)-tolerant citrus cultivars emitted significantly higher levels of certain VOCs, such as green leaf volatiles, thymol, thymol-related derivatives, 4,8-dimethylnona-1,3,7-triene, and 4,8,12-trimethyltrideca-1,3,7,11-tetraene, than HLB-sensitive citrus cultivars when responding to *Candidatus* Liberibacter asiaticus infection (Deng et al., 2021b). Consequently, it seems evident that evaluating the leaf volatile profiles of more citrus genotypes would make a valuable contribution to citrus species identification, chemotaxonomic studies, and understanding the differential tolerance to citrus HLB.

As an efficient and convenient sampling technique (González-Mas et al., 2009; Escriche et al., 2011; Jeleń et al., 2017), static headspace (static-HS) sampling combined with gas chromatography-mass spectrometry (GC-MS) analysis is an ideal method for analyzing plant VOCs (Chen et al., 2015; Mohammadhosseini et al., 2017), facilitating the simple assay of a large number of sample matrices. In addition, compared with HS solid-phase microextraction (HS-SPME), it has the advantages of avoiding costly and time-consuming sample preparation and enables continuous sampling, as the extraction head needs not be replaced between samples (Lorenzo et al., 2002; Yun et al., 2021).

In this study, we aimed to establish a simple and reliable HS-GC-MS technique that could be applied for the extraction and identification the VOCs in citrus leaves. To verify the efficacy of this HS-GC-MS-based method, we used this system to determine volatile compounds in the leaves of 42 citrus cultivars, entailing a qualitative and quantitative comparison of the leaf volatile profiles of the 42 cultivars. Having established the utility of the newly developed method, we performed multivariate statistical analyses, including principal component analysis (PCA) and partial least-squares discriminate analysis (PLS-DA), to evaluate the possibility of discriminating amongst these citrus cultivars on the basis of their leaf volatile profiles. Analysis based on a combination of variable importance in projection (VIP) within the PLS-DA model and one-way analysis of variance (ANOVA) was conducted to identify candidate volatile biomarkers that could be used for discriminatory purposes. The method developed in this study provides a new, rapid, simple, and reliable approach for the extraction and identification of citrus leaf VOCs, and based on this methodology, we propose a leaf volatile profile-based classification model for citrus.

## Materials and methods

### Plant materials

A total of 42 citrus cultivars of different origin were grown at Jiangmiao Village, Jiang’an County, Yibin City, Sichuan Province, China (longitude, 105˚07′E; latitude, 28˚72′N) under identical climatic conditions and horticultural practices. The selected cultivars were of species in the orange and mandarin groups, which represent the most commonly cultivated citrus groups in China. Cultivar specifications are presented in **Table 1**. In total, we collected 12 mature leaves from trees of the same size, at a similar maturation stage, and with no obvious evidence of diseases or insect pest infestation. The leaves were collected randomly from different orientations in the top, middle, and bottom of canopy layers. For each citrus cultivar, three biological replicates were prepared, with each biological replicate comprising leaves collected from three trees. Samples were stored in a cold chamber and transported to the laboratory within 2 h. Upon arrival at the laboratory, the samples were washed with running water, frozen using liquid nitrogen, and stored at -80 ˚C until further analysis.

**Table 1:** Citrus cultivars used in this study.

No.	Common name	Scientifific name	Abbreviation
1	Late-maturing No.8 blood orange	*Citrus sinensis* L. Osbeck	L8BO
2	Tarroco new Line blood orange	*Citrus sinensis* L. Osbeck	TNLBO
3	Chang Ye Xiang Cheng	*Citrus sinensis* L. Osbeck	CYXC
4	Qing Qiu navel orange	*Citrus sinensis* L. Osbeck	QQNO
5	Lun Wan navel orange	*Citrus sinensis* L. Osbeck	LWNO
6	Cara Cara navel orange	*Citrus sinensis* L. Osbeck	CCNO
7	Ooita wase	*Citrus reticulata* Blanco	OW
8	Tsunokaori (Tangor Norin No. 3)	*Citrus reticulata* Blanco	Tsunokaori
9	Ehime Kashi No.14	*Citrus reticulata* Blanco	EK14
10	Gold Nugget mandarin	*Citrus reticulata* Blanco	GNM
11	Or	*Citrus reticulata* Blanco	Or
12	Nichinan No.1	*Citrus reticulata* Blanco	Nichinan1
13	Setoka	*Citrus reticulata* Blanco	Setoka
14	Setomi	*Citrus reticulata* Blanco	Setomi
15	Yellow-peel Shiranui	*Citrus reticulata* Blanco	YShiranui
16	Himeruby	*Citrus reticulata* Blanco	Himeruby
17	Hong Yun xiang gan	*Citrus reticulata* Blanco	HYXG
18	Kanpei	*Citrus reticulata* Blanco	Kanpei
19	Okitsu No.58	*Citrus reticulata* Blanco	Okitsu58
20	W. murcott	*Citrus reticulata* Blanco	Wmurcott
21	USA sugar mandarin	*Citrus reticulata* Blanco	USASM
22	Jinqiu Shatangju	*Citrus reticulata* Blanco	JQSTG
23	Jinkui Shatangju	*Citrus reticulata* Blanco	JKSTG
24	Murcott	*Citrus reticulata* Blanco	Murcott
25	Ehime Kashi No.38	*Citrus reticulata* Blanco	EK38
26	Daya gan	*Citrus reticulata* Blanco	Daya
27	Seedless Or	*Citrus reticulata* Blanco	SOr
28	Himekoharu	*Citrus reticulata* Blanco	Himekoharu
29	Ehime Kashi No.28	*Citrus reticulata* Blanco	EK28
30	Kinnow	*Citrus reticulata* Blanco	Kinnow
31	Fortune	*Citrus reticulata* Blanco	Fortune
32	Sweet Spring tangelo	*Citrus reticulata* Blanco	SST
33	Pixie	*Citrus reticulata* Blanco	Pixie
34	Tsunonozomi (Mikan Norm No. 18)	*Citrus reticulata* Blanco	Tsunonozomi
35	Haruka	*Citrus reticulata* Blanco	Haruka
36	Hassaku	*Citrus reticulata* Blanco	Hassaku
37	Ehime Kashi No.36	*Citrus reticulata* Blanco	EK36
38	Qinju No.1	*Citrus reticulata* Blanco	QJ1
39	Okitsu No.60	*Citrus reticulata* Blanco	EK60
40	Mihaya	*Citrus reticulata* Blanco	Mihaya
41	Kiyomi	*Citrus reticulata* Blanco	Kiyomi
42	BeniBae	*Citrus reticulata* Blanco	BeniBae

### Chemicals and reagents

Reference compounds, including *n*-alkane (C_7_-C_40_) standards, *n*-hexanol, and other available authentic standards, the purities of which were greater than 99.0%, were purchased from Sigma-Aldrich (St. Louis, MO, USA). Ultrapure water was prepared using a Milli-Q water purification system (Millipore Corporation, Bedford, MA, USA) equipped with a 0.22-μm filter.

### Volatile extraction

The leaves of each biological replicate were pooled and fully ground in liquid nitrogen using a mortar and pestle to yield a fine powder. For each extraction sample, 1 g of finely powdered sample was added to a 20-mL HS vial (Thermo Scientific, Bellefonte, PA, USA) containing 0, 1, and 5 mL of saturated sodium chloride solution (NaCl) and 30 μL of an internal standard (0.1% *n*-hexanol). The vial was sealed with an aluminum crimp cap with a PTFE/silicon septum (Millipore Sigma, St. Louis, MO, USA). The sealed vials were mixed thoroughly prior to being placed on a static 7697A HS auto-sampler (Agilent Technologies, Inc., Santa Clara, CA, USA), awaiting injection.

### Static headspace sampling conditions

For the purposes of heating, we used four oscillator temperatures (40, 60, 80, and 100 ˚C). Based on our previous experience, we select quantitative loop and transfer line temperatures that were 10 and 20 ˚C higher than the heating oscillator temperatures, respectively. Accordingly, the quantitative loop temperatures were set to 50, 70, 90, and 110 ˚C and the transfer line temperatures were set to 60, 80, 100, and 120 ˚C. The vial equilibration times were 15 and 30 min,the injection time was 0.5 min, the quantitative loop volume was 0.5 μL, and the constant flow was 15 psi.

### Gas chromatography-mass spectrometry conditions

The quantitative analysis of volatile metabolites was carried out using an Agilent 7890A GC system coupled with a 5975C inert mass selective detector (MSD) with a Triple-Axis detector (Agilent Technologies Inc.). Using a split mode (50:1), each sample (0.5 μL) was injected at 250 ˚C onto an HP-5 MS capillary column (0.25 mm × 30 m; 0.25 μm film thickness; Agilent Technologies Inc.). The column oven temperature was programmed according to our previously published protocol (Deng et al., 2021b) with a minor modification. Analytes were propelled by helium (99.999% purity) at a constant flow rate of 1.0 mL/min. Mass spectra in the electron impact mode (MS-EI) were obtained at an ionization energy of 70 eV. The quadrupole, MS source temperature, and transfer temperatures were set at 150, 230, and 250 ˚C, respectively. Data acquisition was performed by scanning ion mass fragments from 35 to 350 m/z with seven scans per second. For determination of the retention index (RI), a commercial hydrocarbon mixture of *n*-alkanes (C_7_−C_40_) was initially run under identical GC-MS programmed conditions.

### Compound identification and quantification

The raw GC-MS data were initially processed using Aglient G1701EA MSD Productivity ChemStation software (Agilent Technologies Inc.). Volatile compounds were identified by comparing the obtained retention indices and mass spectrograms with those of authentic standards. In the case of any absent reference standards, volatile compounds were tentatively identified based on comparison with retention indices and mass spectrograms previously reported in the literature or archived in online databases, including the National Institute of Standards and Technology library (NIST, Gaithersburg, MA, USA), Flavornet (http://www.flavornet.org/flavornet.html), Pherobase (http://www.pherobase.com), and PubChem (https://pubchem.ncbi.nlm.nih.gov/) databases. Relative concentrations of the target volatile compounds were calculated based on their respective peak areas relative to those of the corresponding internal standards.

### Data processing and statistical analysis

All data are presented as means ± standard deviation of at least three replicates. One-way analysis of variance (ANOVA) and Tukey’s honestly significant difference (HSD) test (*P* < 0.05) were applied to compare variances of the leaf volatile profiles of different cultivars. The soft independent modeling of class analogy (SIMCA) software v. 16.1 (Umetrics, Umea, Sweden) was employed for multivariate statistical analyses (PCA and PLS-DA), prior to which, the data underwent normalization based on the sum of the samples and other default settings to minimize interference (De Livera et al., 2012). The variable importance in the projection (VIP) score provides a measure of the contribution of each x variable for each x variate in the PLS-DA prediction model. In this study, the variables of importance were selected based on VIP scores greater than 1. The PLS-DA model was validated by running 100 permutation tests. Hierarchical cluster analysis (HCA) and heatmap construction were implemented using the JMP Pro 14.1 software (SAS Institute Inc. Cary, NC, USA) using the Ward’s hierarchical clustering method.

## Results and discussion

### Optimization of static headspace sampling parameters

HS techniques, including static and dynamic HS, HS sorptive extraction, solid-phase microextraction (SPME), and direct thermal desorption, are robust methods that facilitate the extraction of volatile compounds in the absence of solvent or any restrictive circumstance that could potentially impede the extraction of pure volatiles from the analyzed samples. Static HS is a convenient, and esay-to-operate automated sampling method (Jeleń et al., 2017), in which the amount of sample, added NaCl, headspace heating temperature, and equilibration time are the most influential factors affecting the accuracy and reliability of volatile extraction (González-Mas et al., 2009; Escriche et al., 2011; Jeleń et al., 2017). For the purpose of the present study, sample weights were empirically selected (Deng et al., 2021b).

The addition of salt in the extraction process has generally been recognized to facilitate the transfer of VOCs from the matrix to the HS *via* a salting-out effect (Risticevic et al., 2010). However, Turazzi et al. (2017) established that saturated NaCl solution was not required for a higher efficiency when using HS-SPME for extraction of pineapple fruit samples. The use of salt to optimize volatile extraction has been well studied in HS-SPME, with findings indicating that an optimal efficacy static HS is obtained with a limited application of salt. Thus, in the present study, we sought to assess the effects of added salt on the efficiency of the HS extraction efficiency of volatile compounds.

**Figure 1:**
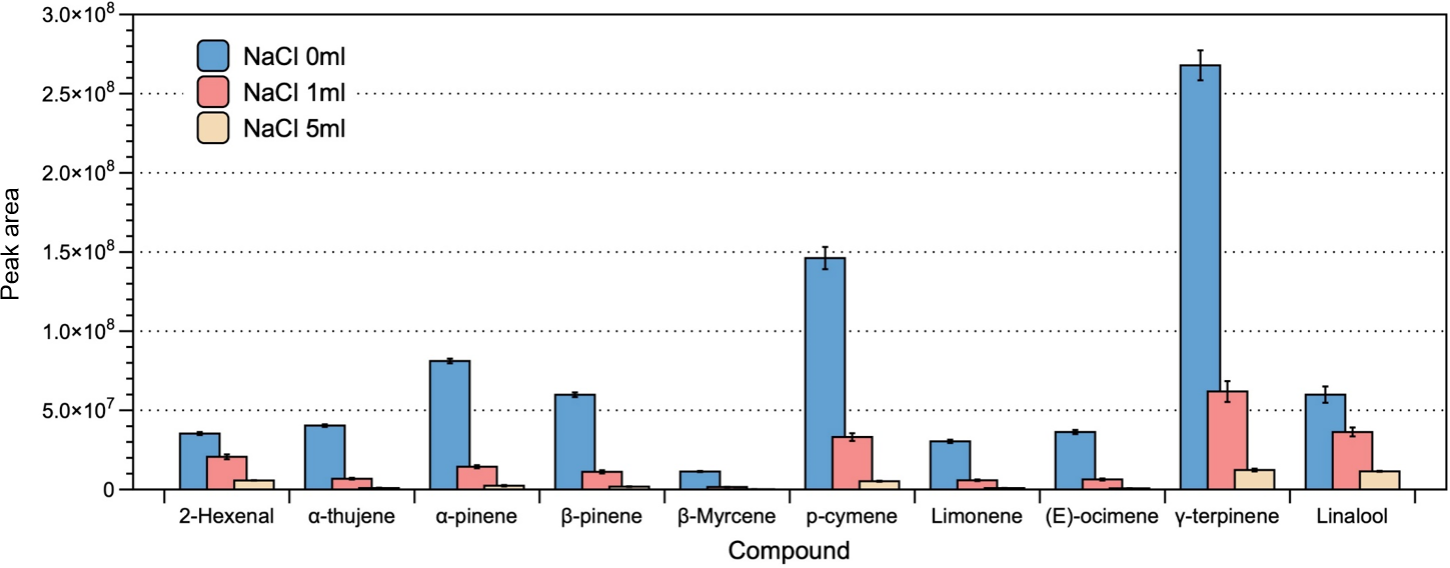
The effect of sodium chloride addition on the efficiency of headspace extraction of volatile compounds. The bars and error bars respectively denote the means and 95% confidence intervals of three biological replicates. Compounds on the x-axis are listed in order of their sequence of elution, with corresponding peak areas shown on the y-axis.

The accuracy and reliability of volatile analyses were evaluated based on the number of peaks and the normalized peak areas for the 10 most predominant volatile compounds. Notably, we found that the largest number of peaks (41) was obtained under salt-free extraction conditions f, followed by the addition of 1 and 5 mL of saturated NaCl (32 and 21 peaks, respectively). Thus, the best results were obtained with extraction in the absence of saturated NaCl solution, for which the peak area was significantly highest (**Figure 1**). Based on these findings, we accordingly decided to not add saturated NaCl solution in further analyses.

**Figure 2:**
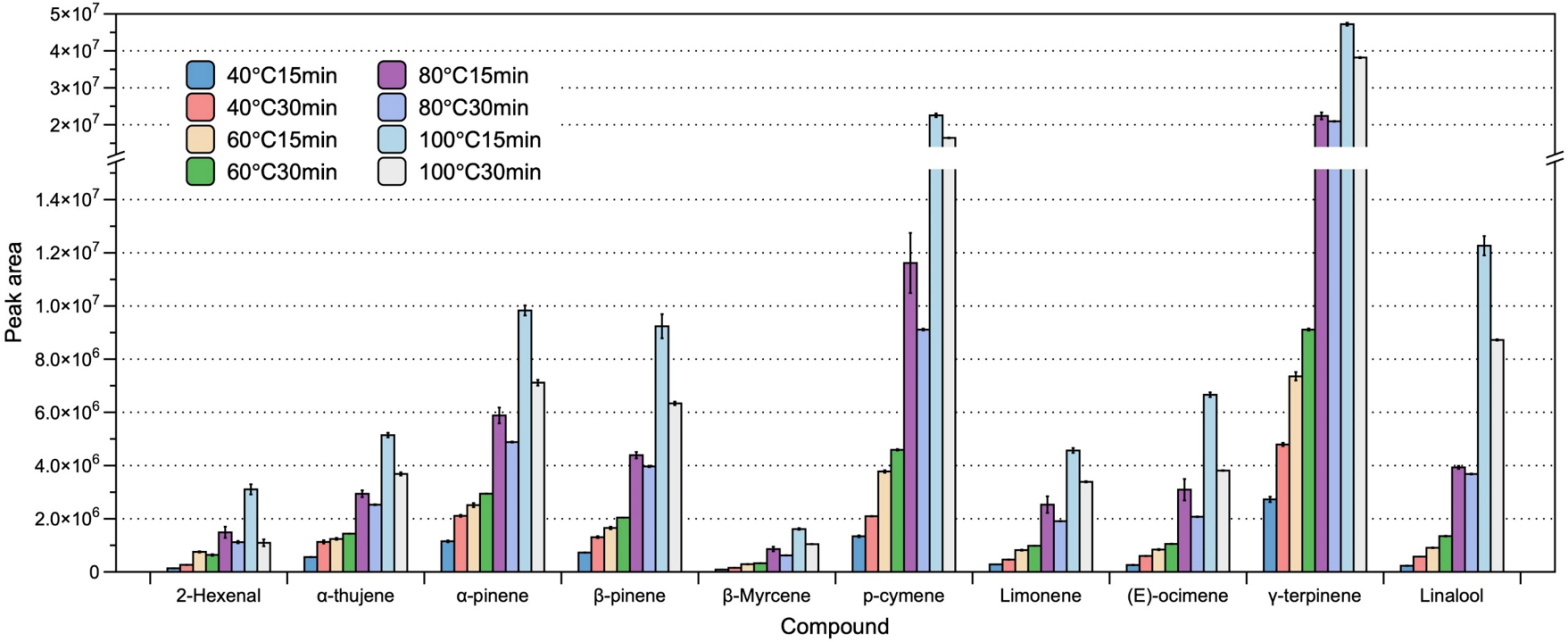
The effect of heating temperature and incubation time on the efficiency of headspace extraction of volatile compounds. The bars and error bars respectively denote the means and 95% confidence intervals of three biological replicates. Compounds on the x-axis are listed in order of their sequence of elution, with corresponding peak areas shown on the y-axis.

In addition to the presence of NaCl, HS heating temperature and incubation time have a pronounced influence on the efficiency of extraction. In the present study, we optimized the heating temperature and incubation time by performing a series of assessment, in which we applied a range of conditions, selected based on our preliminary experiments and previous studies. Specifically, we examined the influence of four commonly used HS heating temperatures (40, 60, 80, and 100 ˚C) and two HS heating times (15 and 30 min). We refrained from assessing temperatures higher than 100 ˚C to avoid the potential degradation of certain volatile analytes. We accordingly found that the extraction efficiency increased with rising temperature, which facilitated the volatilization of compounds from the matrix to the overlying HS. With respect to incubation time, we established that at low heating temperatures (40 and 60 ˚C), prolonging the incubation time (from 15 to 30 min) resulted in increases in the amounts of extracted compounds. However, we could not detect higher volatile concentration when increasing the incubation time from 15 to 30 min when heating at the higher temperatures of 80 and 100 ˚C. Based on this assessment of different combinations of HS heating temperature and incubation time, we established that the optimal HS extraction conditions were incubation for 15 min at 100 ˚C (**Figure 2**).

### Application of the optimized HS-GC-MS method for determining the leaf volatile profiles of 42 citrus cultivars

**Figure 3:**
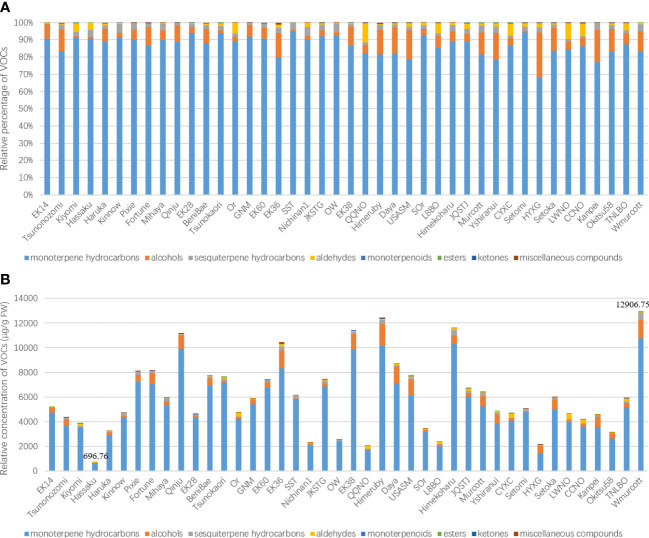
Relative percentages of different volatile chemical categories **(A)** and the relative total concentration of volatile metabolites **(B)**.

To evaluate the potential application of the optimized HS-GC-MS method in the analysis of different citrus genotypes, we used this system to determine the leaf volatile constituents of 42 citrus cultivars. In total, 83 compounds were identified among the leaf volatile profiles of the 42 citrus cultivars based on comparisons with authentic standards, previous literature, and online databases. The average number of leaf volatile metabolites per cultivar was 47, ranging from 33 constituents in Hassaku to 62 in Setomi (**Table S1**). Representative chromatographic profiles of selected cultivars are shown in **Figure S1**. These VOCs comprised a complex combinations of different classes of coumpound, with monoterpene hydrocarbons being identified as the predominant constituent group (68.23%–95.08%, 21 compounds), followed by alcohols (0.69%–26.0%, 8 compounds), sesquiterpene hydrocarbons (0.47%–5.04%, 26 compounds), aldehydes (0.12%–11.26%, 10 compounds), monoterpenoids (0%–0.36%, 7 compounds), esters (0%–0.18%, 5 compounds), ketones (0%–0.02%, 2 compounds), and miscellaneous compounds (0%–1.11%, 4 compounds) (**Figure 3A**). This chemical classification is consistent with the major constituents of citrus volatiles that have previously been identified (Azam et al., 2013; Matsukawa et al., 2017; Lamine et al., 2018; González-Mas et al., 2019; Deng et al., 2021b).

The total relative concentrations of leaf VOCs of the assessed citrus cultivars differed considerably, particularly in the case of mandarins (*C. reticulata* Blanco). Totals of 696.76 ± 25.06 and 12906.75 ± 315.69 μg/g fresh weight (FW) relative concentration were detected in the leaf VOCs of the cultivars Hassaku and W. Murcott, respectively. Compared with oranges, mandarins contained a relatively higher amount of average total volatile content (6368.98 ± 907.05 μg/g FW), with former having an average content of 3964.44 ± 596.44 μg/g FW, ranging from 2029.45 ± 67.15 μg/g FW in Qin Qiu navel orange to 5897.14 ± 160.01 μg/g FW in Tarroco new line blood orange (**Figure 3B**).

### Comprehensive metabolic fingerprinting of the leaf volatiles in 42 citrus cultivars

Metabolic fingerprinting involves comparing the metabolomes of two or more systems to determine whether they are biologically related. One of the hallmarks of metabolic fingerprinting is the use of multivariate statistics in the processing of data to pinpoint spectral features that are biologically relevant for further investigation (Worley and Powers, 2013). Two of the most widely applied multivariate statistical techniques are the unsupervised PCA and supervised PLS-DA analyses, which can respectively employed to generate visual interpretations of the correlations and variability of highly complex data sets (Vidal et al., 2016), and to optimize the separation between different samples and identify potential biomarkers (Kalivodová et al., 2015).

**Figure 4:**
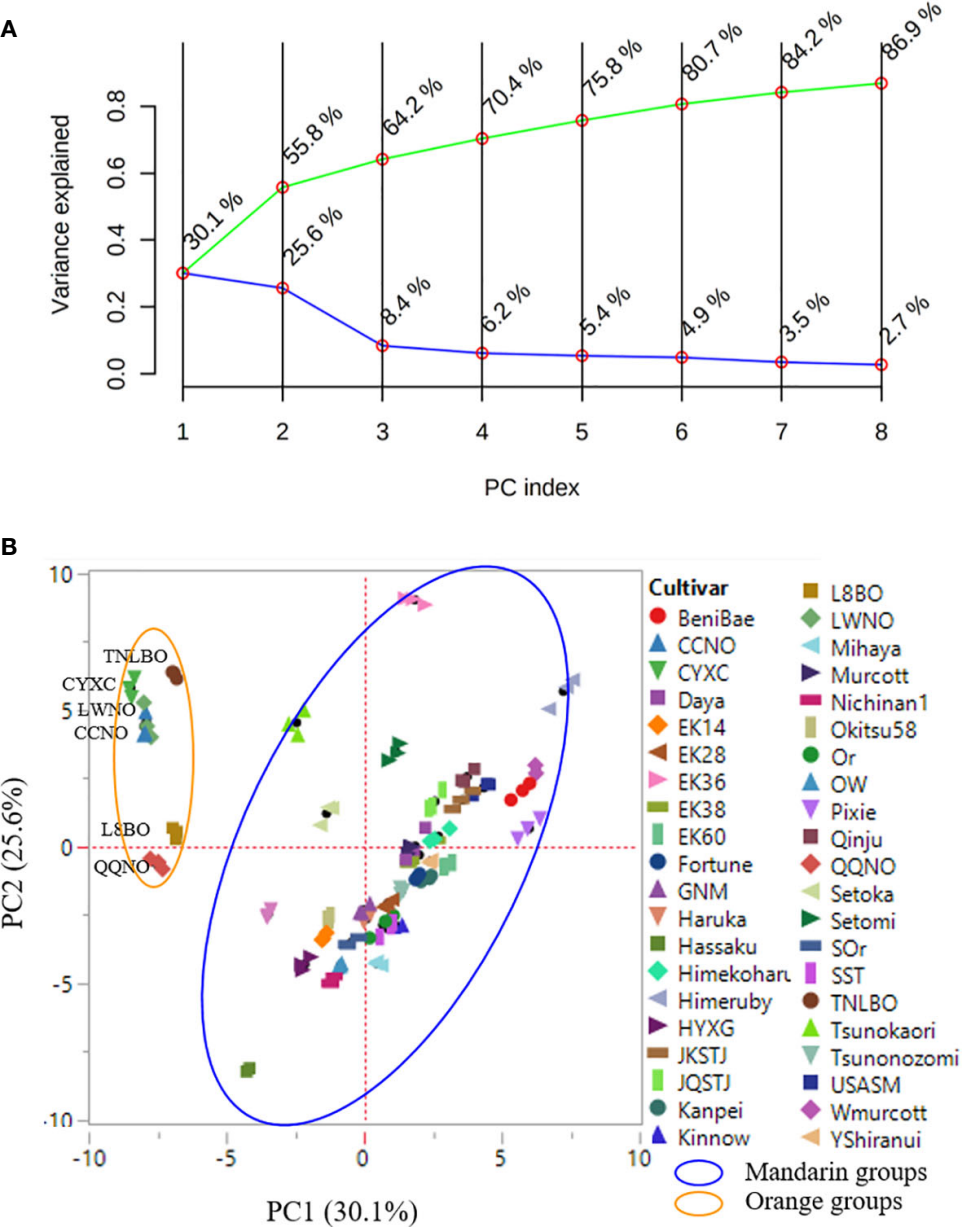
Principal component analysis (PCA) scree plot **(A)** and score plot **(B)**. Data points representing three biological replicates are ploted in close proximity, and in **(B)** the mandarin and orange group cultivars can be seen to form distinct clusters. Values in parentheses along the axes indicate the variance explained.

The PCA model adopted in the present study was found to show excellent goodness-of-fit and predictive ability with R^2^X_(cum)_ and Q^2^_(cum)_ values of 0.89 and 0.60, respectively. The first eight principal components (PCs) were established to contain approximately 87% of the feature variance amongst the 42 citrus cultivars, whereas the variance contribution rates of the first and second PCs were 30.1% and 25.6%, respectively (**Figure 4A**). The spatial distribution of the first two PCs showed clear separation between the mandarin and orange groups, consistent with their genetic profiles. Moreover, the data points for the three biological replicates of each citrus cultivar were typically superimposed or were at least plotted in close proximity, thereby indicating the excellent repeatability of the experiments. Orange group cultivars negatively correlated with PC1, whereas most of these, with the exception of Qingqiu navel orange, positively correlated with PC2. Although approximately grouped, the cultivars in the mandarin groups displayed a complex spatial distribution with respect to the first two PCs, which tends to be indicative of the complex nature and diversity of the mandarin group cultivars (**Figure 4B**). However, in general, the PCA model could not effectively distinguish mandarin cultivars based on their leaf volatile profiles.

**Figure 5:**
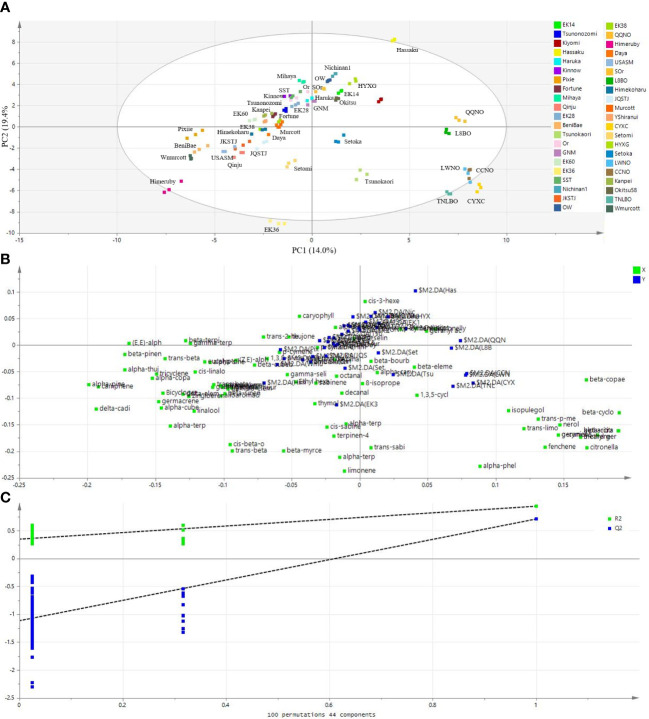
Partial least-square discriminate analysis (PLS-DA) showing a better separation amongst the 42 citrus cultivars based on their leaf volatile profiles. **(A)** A score plot of the PLS-DA results, **(B)** a loading plot of the PLS-DA results, and **(C)** statistical validation of the PLS-DA model based on permutation tests (100 permutations; 44 components). The green squares in **(B)** represent the volatiles that contributed to the discrimination of citrus cultivars (blue squares).

Given that PCA could not clearly separate the mandarin cultivars (**Figure 4**), as an alternative approach, we performed PLS-DA. Notably, the PLS-DA score plot showed a better discrimination than the PCA amongst the 42 citrus cultivars (**Figure 5A**). In the contest of the PLS-DA model, the values of R^2^X_(cum)_ and R^2^Y_(cum)_ represent the goodness-of-fit, and the parameter Q^2^_(cum)_ was used to assess the predictive ability of the model (Kalivodová et al., 2015). The corresponding values of the parameters of R^2^X_(cum)_, R^2^Y_(cum)_, and Q^2^_(cum)_ were 0.998, 0.956, and 0.801, respectively, indicating that the model employed was valid and robust with excellent goodness-of-fit and good predictive accuracy. The total amounts of variation explained by PC1, PC2, and PC3 were 14.0%, 19.4%, and 17.1%, respectively (**Figure 5A**).

There are also several other parameters assessed using the PLS-DA model, such as loading weight and VIP, that might be useful in identifying the most important variables (Kalivodová et al., 2015). **Figure 5B** shows a corresponding loading plot in which both variables (volatile metabolites) and products (citrus cultivars) are plotted on the same plane. Validation of the supervised PLS-DA model was performed by running 100 permutation tests. This permutated model gave the intercepts of R^2^ = (0.0, 0.346) and Q^2^ = (0.0, -1.03) (**Figure 5C**), representing the explained variance and the predictive capability, respectively (Deng et al., 2021a), and indicating the robustness of the PLS-DA model.

**Figure 6:**
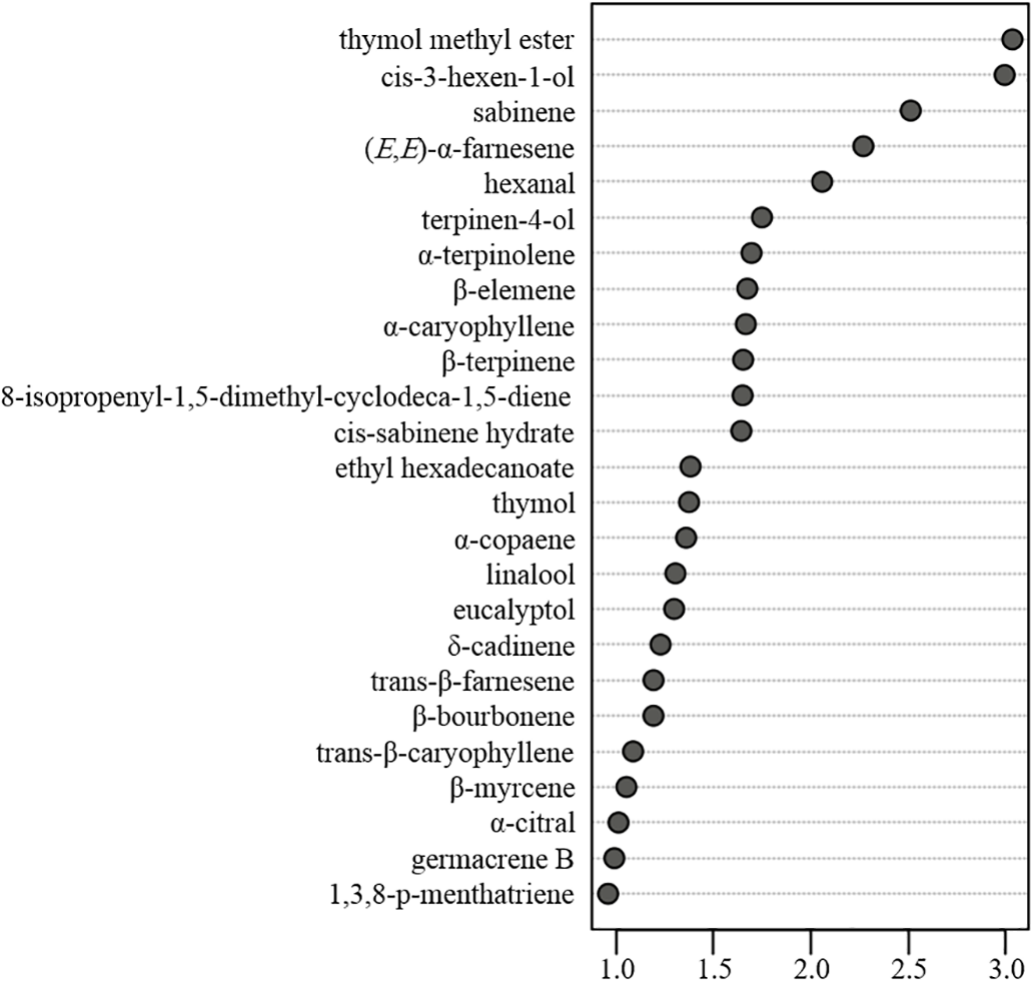
The most relevant characterized volatiles ranked by variable importance in projection score.

VIP values can reflect the contribution of individual compounds in discriminating among the products. In this regard, variables with a higher VIP score based on PLS-DA are considered to be more relevant (Chong and Jun, 2005). In this study, we identified a total of 25 volatile compounds with a VIP score > 1.0 and p value < 0.05 (graphically presented in **Figure 6**), indicating that these are the most important among the characterized volatile compounds with respect discriminating amongst the assessed citrus cultivars. Among these compounds, *cis*-sabinene hydrate, sabinene, thymol, and thymol methyl ether are involved in the biosynthetic pathway of thymol and thymol-related derivatives, the defensive roles of which were confirmed in our previous study (Deng et al., 2021b). (*Z*)-3-hexen-1-ol, also known as leaf alcohol, is among the main sources of the characteristic green aroma and has important allelopathic effects on plants, and natural enemies (Kessler and Baldwin, 2001). Hexanal, an aliphatic aldehyde, is among the self-aldol condensation productsthat have desirable organoleptic qualities, as determined by aroma and taste evaluations in *Citrus* (González-Mas et al., 2019).

**Figure 7:**
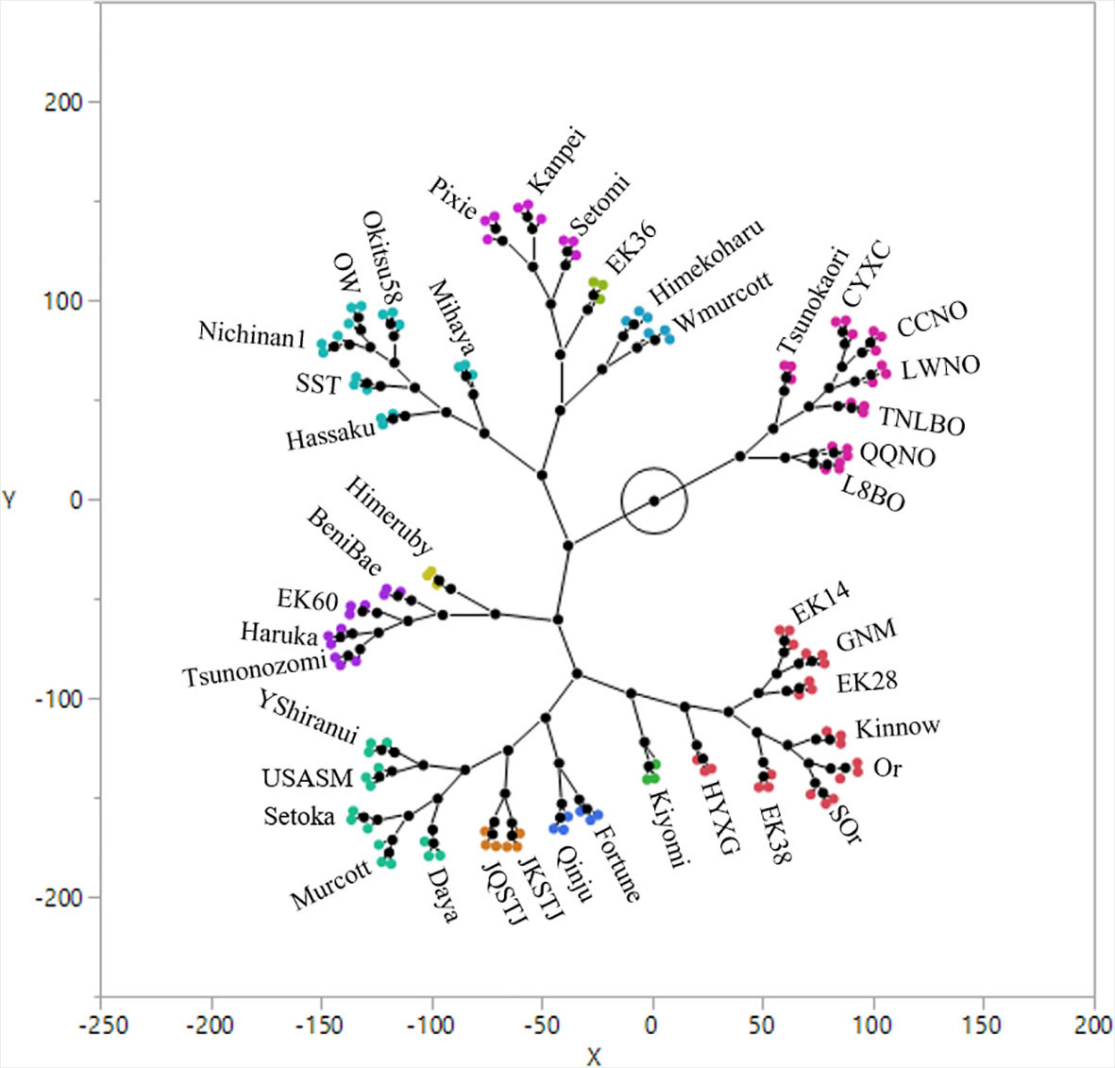
Hierarchical cluster analysis of the 42 citrus cultivars based on their leaf volatile profiles.

HCA confirmed that the mandarin and orange groups have notably differential volatile profiles, with the mandarin group generally being characterized by volatile profiles of great complexity (**Figure 7**). The results of a two way clustering (clusters with columns and rows) of sample and compound scales are shown in **Figure S2**. The Tsunokaori tangor cultivar, was obtained by hybridization of Kiyomi tangor (*C. sinensis* × *C*. *unshiu*) × Okitsu wase (*C*. *unshiu*), has an orange aroma and a peel that can be moderately adherent (Matsumoto et al., 1991). In the present study, we established that its leaf volatile profile is more similar to that of cultivars in the orange groups. Based on the leaf volatile profiles obtained in this study, the assessed mandarin cultivars can be grouped into five clusters, with some sub-clusters (**Figure 7**). The constellation plot shown in **Figure 7** presents direct visual evidence indicating the closeness of the leaf volatile profiles of the 42 assessed citrus cultivars.

## Conclusions

In this study, we developed and optimized an efficient and convenient static headspace sampling technique combined with gas chromatographic-mass spectrometic analyses for the determination of the leaf volatile profiles of citrus cultivars. The validation on a large set of 42 cultivars provided confirmation of the applicability of this optimized HS-GC-MS system in determining leaf volatile profiles. Based on multivariate data analysis, we identified the most important of the characterized volatile compounds with respect to discriminating amongst the citrus cultivars. HCA was used to categorize the citrus cultivars according to similarities and differences in their volatile compounds.

## Data availability statement

The original contributions presented in the study are included in the article/**Supplementary Material**. Further inquiries can be directed to the corresponding author.

## Author contributions

HD and ZW conceived and planned the experiment. RMH, RH, CP, YM, HX, and DL performed the whole experiment. HD, RMH, LL, BX, XW, and MZ participated in and performed statistical analysis. RMH, XA, BY, DH, and HD participated in plant materials preparation. HD and RMH wrote the original draft manuscript. HD revised and edited the manuscript. All authors have read and approved the final manuscript.
