# Supplementary material for: Optimization of a static headspace GC-MS method and its application in metabolic fingerprinting of the leaf volatiles of 42 citrus cultivars
Source: Front Plant Sci. 2022 Dec 8;13:1050289. doi: 10.3389/fpls.2022.1050289 (PMC9772436; doi:10.3389/fpls.2022.1050289)

Figure S1. Representative chromatographic profiles of (A) Nichinan No.1, (B) Lun Wan navel orange, (C) Ehime Kashi No.36, (D) Setomi, and (E) Ehime Kashi No.38.

Figure S2. Heatmap of individual leaf volatile metabolites determined in 42 citrus cultivars. Blue and red color indicate the low and high content of different volatile metabolites, respectively.

**Figure S1**


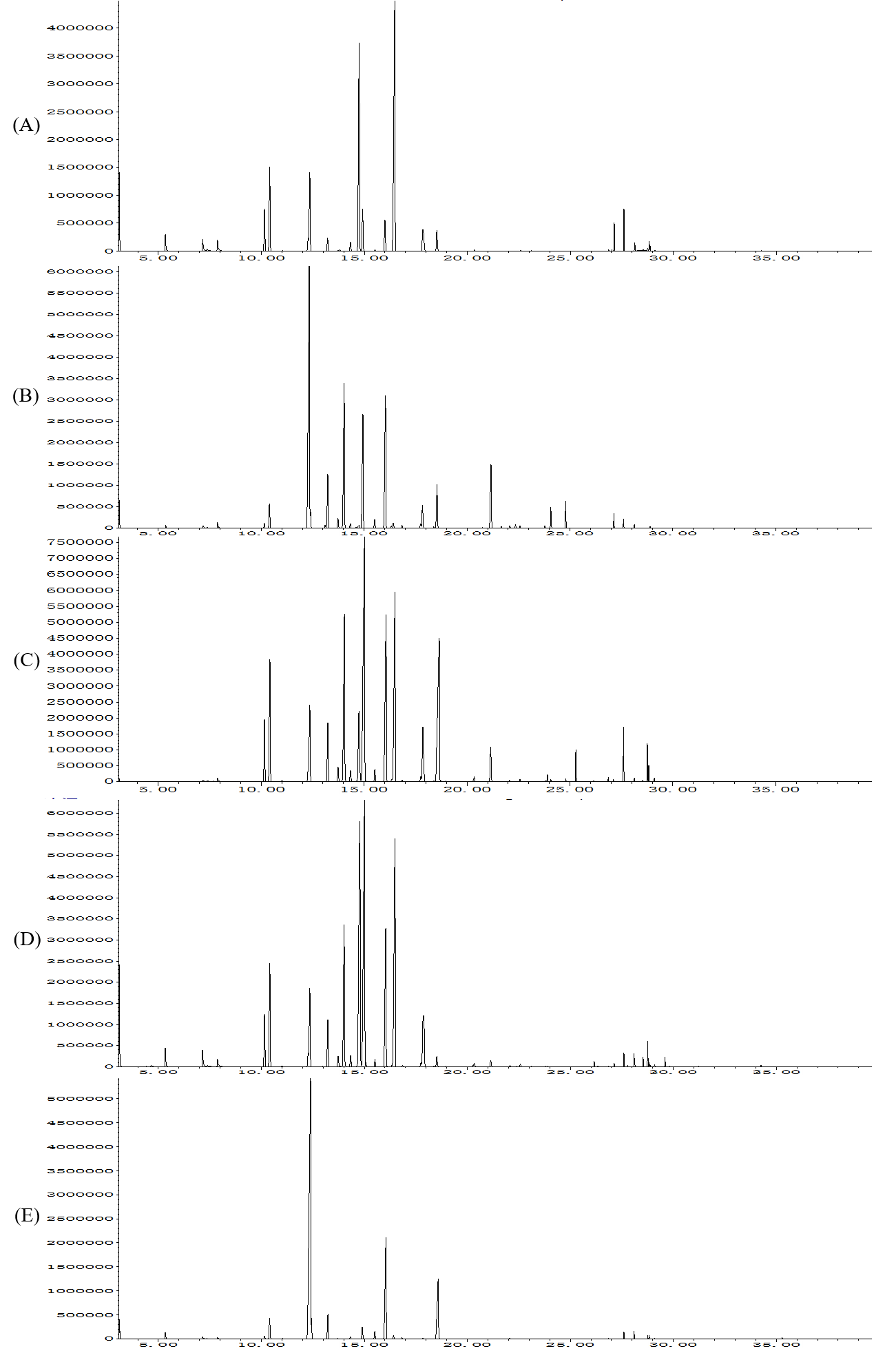


Figure S2


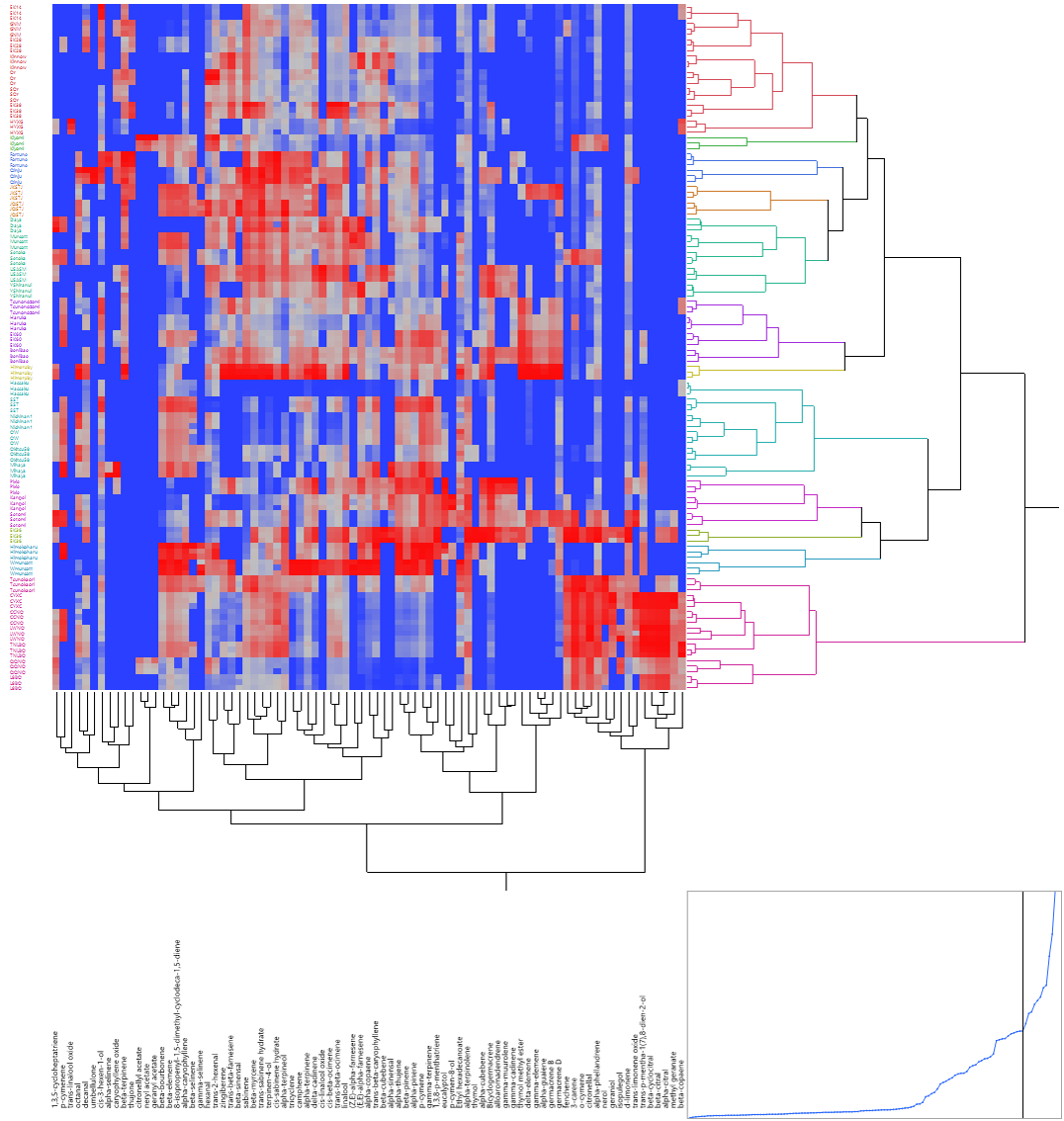

Supplement: Supplementary file 1 [file DataSheet_1.docx]
